# Supplementary material for: The Systems Biology Research Tool: evolvable open-source software
Source: BMC Syst Biol. 2008 Jun 29;2:55. doi: 10.1186/1752-0509-2-55 (PMC2446383; doi:10.1186/1752-0509-2-55)
Supplement: Additional file 1 — SBRT Archive. An archive of the current version of the Systems Biology Research Tool. [file 1752-0509-2-55-S1.zip › sbrt-1.4.0/doc/developers_guide/api/sbrt/shell/mng/ShellProcessManager.html]

ShellProcessManager


|  |  |  |
| --- | --- | --- |
| **Overview** \| **Package** \| **Class** \| **Use** \| **Tree** \| **Deprecated** \| **Index** \| **Help** | |  |
| **PREV CLASS**   NEXT CLASS | **FRAMES**    **NO FRAMES**     **All Classes** |  |
| SUMMARY: NESTED \| FIELD \| CONSTR \| METHOD | DETAIL: FIELD \| CONSTR \| METHOD |  |


---


## sbrt.shell.mng Interface ShellProcessManager<K,V>

**Type Parameters:**: `K` - the key type.: `V` - the value type.

**All Superinterfaces:**: ProcessManager<K,V>

**All Known Implementing Classes:**: BiggSbmlReaderManager, BiggSbmlTranslaterManager, LinearCombConverterManager, MatrixConverterManager, MetatoolFileWriterManager, MultipleVectorsConverterManager, PalssonSbmlReaderManager, PalssonSbmlTranslaterManager, SbmlWriterManager, SimpleRxnFileTransManager, SingleVectorConverterManager, UniqueCycleIdManager

---

``` public interface ShellProcessManager<K,V> extends ProcessManager<K,V> ```

This interface is used to represent shell process managers.
A shell process manager is used to manage a process executed in the shell,
rather than the kernel. The use of this functionality should be limited to processes
that are consistent with the responsibilities of the shell, such as file I/O.
An example shell process would be converting one file format to another. Since the kernel
does not deal with files or parsing text, this is a suitable shell process.

A shell process manager is created from a `ManagerInput`
object. Shell process managers require a minimum set of key-value
pairs to perform their action.
In addition to this minimum set, a set of optional
key-value pairs may also be allowed. If the provided `ManagerInput`
contains any additional keys beyond the set of required and optional keys,
an `ApplicationException` will be thrown.

**Author:**
:   This interface was written and documented by
    Jeremiah Wright while in the Wagner lab.

---

**Method Summary**

**Methods inherited from interface sbrt.shell.mng.ProcessManager**

`check, execute, execute, getOptionalKeys, getRequiredKeys, setInput, setInput`

---


|  |  |  |
| --- | --- | --- |
| **Overview** \| **Package** \| **Class** \| **Use** \| **Tree** \| **Deprecated** \| **Index** \| **Help** | |  |
| **PREV CLASS**   NEXT CLASS | **FRAMES**    **NO FRAMES**     **All Classes** |  |
| SUMMARY: NESTED \| FIELD \| CONSTR \| METHOD | DETAIL: FIELD \| CONSTR \| METHOD |  |


---
